# Know When You Are Too Many: Density-Dependent Release of Pheromones During Host Colonisation by the European Spruce Bark Beetle, *Ips typographus* (L.)

**DOI:** 10.1007/s10886-023-01453-y

**Published:** 2023-10-03

**Authors:** Tobias Frühbrodt, Baoguo Du, Horst Delb, Tim Burzlaff, Jürgen Kreuzwieser, Peter H. W. Biedermann

**Affiliations:** 1 Department of Forest Protection, Forest Research Institute Baden-Württemberg, Wonnhaldestrasse 4, 79100 Freiburg, Germany; 2 Chair of Ecosystem Physiology, University of Freiburg, Georges-Köhler-Allee 53, 79110 Freiburg, Germany; 3 Chair of Forest Entomology and Protection, University of Freiburg, Fohrenbühl 27, 79252 Stegen-Wittental, Germany

**Keywords:** Verbenone, Verbenol, Aggregation inhibitor, Host-marking pheromones, Colonisation density, Intraspecific competition

## Abstract

Individuals across various animal species communicate their presence to conspecifics. Especially phytophagous and parasitoid insects with their brood developing on limited resources rely on chemical cues, such as host-marking pheromones, to reduce intraspecific competition. Bark beetles are phytophagous insects with some species being economically and ecologically relevant forest pests. Several of them use the volatile compound verbenone to inhibit attraction and reduce intraspecific competition. However, in the Eurasian spruce bark beetle, *Ips typographus* (L.), temporal emission patterns did so far not quite support the putative function of verbenone as an indicator of densely colonised host trees. More importantly, it is currently unclear how well verbenone emission is actually related to colonisation density and thus intraspecific competition. Here, we inoculated Norway spruce logs with *I. typographus* at two defined colonisation densities in the greenhouse and measured the emission of verbenone and its precursors α-pinene and verbenol over time. Verbenone emission was 3–7 times greater from colonised logs compared to decaying logs without beetles during the major part of larval development. Furthermore, our data supports the quantitative hypothesis, that the termination of attack on a tree is mediated by a cessation of the release of verbenol and continuous emission of verbenone. The latter is most likely a passively produced host-marking cue reflecting the actual density of conspecifics since per-beetle emission was unaffected by colonisation density. These findings shed new light on the regulation of bark beetle mass aggregations, which are currently causing previously unseen economic damages in temperate forests.

**Supplementary Information:**

The online version contains supplementary material available at 10.1007/s10886-023-01453-y.

## Introduction

Animals across many different taxa communicate their presence to conspecifics to avoid competition, and many do so through chemical cues or signals (Wyatt 2014). Cues convey information to the receiver only incidentally and thus did not evolve for the purpose of communication, whereas signals specifically evolved to convey information from a sender to a receiver (Bradbury and Vehrencamp 2001). Particularly species whose offspring depends on a strongly limited resource need to employ cues or signals that help to avoid overcrowding (Renwick 1989). Behavioural responses towards such cues or signals are therefore common among phytophagous and parasitoid insects, for which the amount of food resources are typically clearly confined (Liu et al. 2011; Nufio and Papaj 2001). Several species thus possess so-called “host-marking pheromones” that are actively produced and released by an individual of a species to signal the presence of brood to conspecifics to reduce intraspecific competition (Nufio and Papaj 2001).

Bark beetles (Curculionidae: Scolytinae) are a group of phytophagous insects that rely on a complex olfactory communication system (Byers 2004). A few bark beetle species are capable of colonising and ultimately killing living host trees (Lindgren and Raffa 2013; Raffa et al. 2015), which gave them exceptional attention due to the economic and ecological damages following populations outbreaks (Gregoiré and Evans 2004; Patacca et al. 2022; Wermelinger 2021). The impacts are likely to be further intensified under the projected increasing temperatures associated with extreme climatic events such as droughts and windthrows: Both positively affect bark beetle populations and negatively impact tree health and consequently their resistance against the beetles (Biedermann et al. 2019; Machado Nunes Romeiro et al. 2022; Patacca et al. 2022). Yet, since trees are not entirely defenceless, but capable to respond to an attack with induced defence (Franceschi et al. 2005), these bark beetle species typically need to mass-aggregate to collectively overcome a tree’s defence (Raffa 2001). For that purpose, the beetles release aggregation-pheromones to attract mates (Silverstein et al. 1966).

The beetles colonising living trees, however, face a dilemma because the attraction of mates also increases the intraspecific competition within a tree (Raffa 2001). In fact, too high intraspecific competition inside a host tree reduces the fitness of the parental generation and its offspring (Anderbrant et al. 1985; Robins and Reid 1997; Sallé and Raffa 2007). Consequently, behaviour that allows to reduce too high intraspecific competition within a host tree should be selected (Raffa 2001). Bark beetles indeed evolved the capacity to detect and respond to cues that indicate high colonisation density or even evolved signals to warn newly arriving beetles to not colonise an overcrowded tree (Borden 1997). The quantitative hypothesis formulated by Schlyter et al. (1987b) suggests that the shift in landing of newly arriving beetles from densely colonised trees to a neighbouring tree is mediated by a simultaneous effect of the cessation of attraction, and the additional presence of cues or signals that inhibit aggregation.

The volatile compound verbenone has been identified as an aggregation inhibitor in several bark beetle species (Renwick 1967; Renwick and Vité 1969; Rudinsky 1973). It is assumed to indicate poor habitat quality due to intra- and interspecific competition (Byers 1989), as well as high microbial activity and decay of the host tissue (Byers 2004; Lindgren and Miller 2002). Chemically, verbenone is synthesised by the oxidation of host tree-born α-pinene via verbenol (Hunt and Borden 1990).

The Eurasian spruce bark beetle (*Ips typographus* L.), is the most destructive forest pest of Norway spruce (*Picea abies* L.) (Gregoiré and Evans 2004) causing significant economic damages in managed spruce forests (Hlásny et al. 2019, 2021). However, during endemic phases its colonisation is restricted to strongly weakened or already dead trees (Cognato 2015). The aggregation pheromone bouquet of *I. typographus* responsible for secondary attraction consists of *cis-*verbenol, 2-methyl-3-buten-2-ol and ipsdienol at specific composition (Bakke et al. 1977; Schlyter et al. 1987a, b). While 2-methyl-3-buten-2-ol and ipsdienol are synthesised *de novo* by the beetles, *cis*-verbenol is derived from the host defence compound α-pinene (Blomquist et al. 2010; Ramakrishnan et al. 2022), and is thus directly linked to the composition of the food substrate provided by the host tree (Lindström et al. 1989). It is still under debate whether primary attraction alone exists in *I. typographus*, but the host derived compound α-pinene at least synergises the attraction of the aggregation-pheromone (Erbilgin et al. 2007).

Verbenone is also found in *I. typographus* (Ramakrishnan 2022; Xie and Lv 2013). Together with specific concentrations of ipsenol (Birgersson et al. 1984; Xie and Lv 2013), it inhibits the attraction of aggregation-pheromones (Bakke 1981) and these two compounds were thus suggested to regulate intraspecific competition (Vité and Francke 1985). Even though verbenone is known since decades, it is still not clear how well its emission is actually related to colonisation densities and whether it is also an actively produced beetle signal or simply a passive cue originating from degraded substrate (Frühbrodt et al. 2023).

If verbenone was an ideal aggregation inhibitor to avoid overcrowding, one would expect that it is only released from colonised hosts once the beetles have successfully mated. However, the few studies that investigated the occurrence of verbenone in *I. typographus* rather show that verbenone occurs much earlier, i.e., immediately after contact with the host tree inside the beetles’ guts (Birgersson et al. 1984), emitted from individual entrance holes (Birgersson and Bergström 1989) and inside the phloem surrounding the galleries (Leufvén and Birgersson 1987). These studies mostly limited their observations to the first week of colonisation. However, new beetles may arrive several weeks after initial colonisation (Anderbrant et al. 1988; Bentz et al. 1997; Toffin et al. 2018), but we do not know whether verbenone is still emitted at this time. Moreover, in contrast to other bark beetle species, e.g. from the genus *Dendroctonus* (Byers et al. 1984; Grosman et al. 1997)*,* the amounts of verbenone found in *I. typographus* are extremely low (< 5 ng beetle^−1^) (Birgersson et al. 1984; Ramakrishnan 2022). Thus, despite its undoubtable inhibitory effect on beetle aggregation (e.g. Bakke 1981), actual evidence is lacking that verbenone functions as an effective anti-aggregation signal in *I. typographus* that allows the beetles to distinguish densely colonised from decaying but not yet colonised hosts.

In this study, we therefore aim to investigate the emission of verbenone, and its precursor compounds verbenol and α-pinene during the colonisation process by *I. typographus* on Norway spruce under semi-controlled conditions in a greenhouse experiment throughout the development of the filial generation. We inoculated Norway spruce logs at different beetle densities to understand how the colonisation density affects the temporal release patterns of these three volatiles. We hypothesise that 1) both α-pinene and verbenol emissions increase at the beginning of colonisation and differ between high and low colonisation density treatments as these compounds are relevant for primary and secondary attraction, and 2) that verbenone is released later than verbenol and in proportionally greater amounts the higher the beetle density. The latter would suggest that its emission is actively regulated by the beetles in response to increased intraspecific competition. Alternatively, verbenone emission may not be under beetle control with the per-beetle emission of verbenone being independent of our beetle density treatments.

## Methods and Material

### Bark beetles, spruce logs and greenhouse

Vital *I. typographus* individuals originating from the Black Forest (near Freiburg, SW Germany, ~ 800–1000 m a.s.l.) were reared on Norway spruce logs in the greenhouse. The experimental beetles were approximately from the F3-F5 generation and freshly emerged 1–3 days prior to the start of the experiment. They were stored at 5°C on moist cellulose tissue until usage. Logs from Norway spruce (length: 60 cm, diameter: 24 ± 4 cm) were obtained from two tree-fellings (near Freiburg, SW Germany, ~ 300–350 m a.s.l.) in October and November 2021 and stored outside, but sheltered from precipitation and soil moisture until usage.

The experiment was conducted between February and April 2022 in a greenhouse under long day conditions (16:8 h, light:dark) with artificial illumination (SIGNION duro, E40, 400W, BLV Licht- und Vakuumtechnik GmbH, Steinhöring, Germany) in addition to the natural daylight. Temperature was not controlled but recorded every 15 min (UA-001–64 HOBO®, Onset, Bourne, USA). It followed a clear circadian rhythm, with an average of 22.9°C and a range from 16.2°C at night to 45.6°C during the day. Daily maximum temperature was most often reached between 14:00–15:00 and minimum temperature between 05:00–06:00. During the times of volatile sampling (approximately 11:00–13:00), mean temperature amounted to 27.2 ± 2°C and relative air humidity to 33.6 ± 10.6%.

### Treatments and beetle inoculation

Five groups of three logs from the same out of four tree individuals were positioned next to each other. Each log within a group from the same tree individual was assigned one of the three treatments: I) untreated negative control without beetles, II) low colonisation density (“low-density”) and III) high colonisation density (“high-density”). The design was completely balanced except that for the two groups originating from the same tree individual only one shared control log was implemented, and one of the logs in the low-density treatment had no successful bark beetle colonisation at the end of the experiment and was discarded from subsequent analysis.

The experiment started on Feb 21^st^, 2022 (day 0). On each log, a 150 × 90 mm area of undamaged bark without branches was defined for the volatile measurements. Within that area, no (control), two (low-density) or nine (high-density) 2-mL-Eppendorf tubes with their bottom cut open were stapled to the bark with two small stainless pins per tube. Because pinning causes minor bark injuries, all treatments received the same number of pins (2 × 9 = 18) also in case when no (control) or fewer tubes (low-density) were needed. Each tube then received five beetles of unknown sex, resulting in a total of 2 × 5 = 10 beetles and 9 × 5 = 45 for the low and high-density treatment, respectively. The high-density group received 24 additional tubes outside the measured area, i.e., 24 × 5 = 120 beetles, to generate intraspecific competition also from outside the measured area. Hence, the nominal infestation density within the measured area was 1.8 and 8.1 entrance holes dm^−2^ for the low and high-density treatment, respectively. The tubes containing beetles were covered with small pieces of insect mesh (0.8 × 0.8 mm, GLAESER grow, Ulm, Germany) to prevent the beetles from escaping. To monitor the developmental stage of the filial generation, three additional logs were inoculated at the same time as the experimental logs. Small segments of bark were arbitrarily selected and removed each time volatiles were sampled, and the developmental stage of the brood inside the phloem was recorded qualitatively.

### Verification of beetle performance and development

On day 1 (approximately 20 h after beetle inoculation) and day 2 all tubes were checked for boring dust and fresh beetles were added in case no activity could be observed in a tube and/or all beetles had visibly died. On day 4 we assumed the construction of nuptial chambers to be terminated, so all tubes were removed from the measured area and the dead beetles on the bark surface outside the phloem were counted and removed.

At the end of the experiment (day 49), the bark inside the measured areas was cut from the logs with some buffer. For each bark sample, the number of entrance and exit holes, maternal and larval galleries, as well as pupal chambers was recorded. The area of consumed phloem was measured. The counting and area measurement were done on scaled photographs using ImageJ (Rasband 1997–2018) and using the original bark sample.

### Volatile sampling

**Fig. 1:**
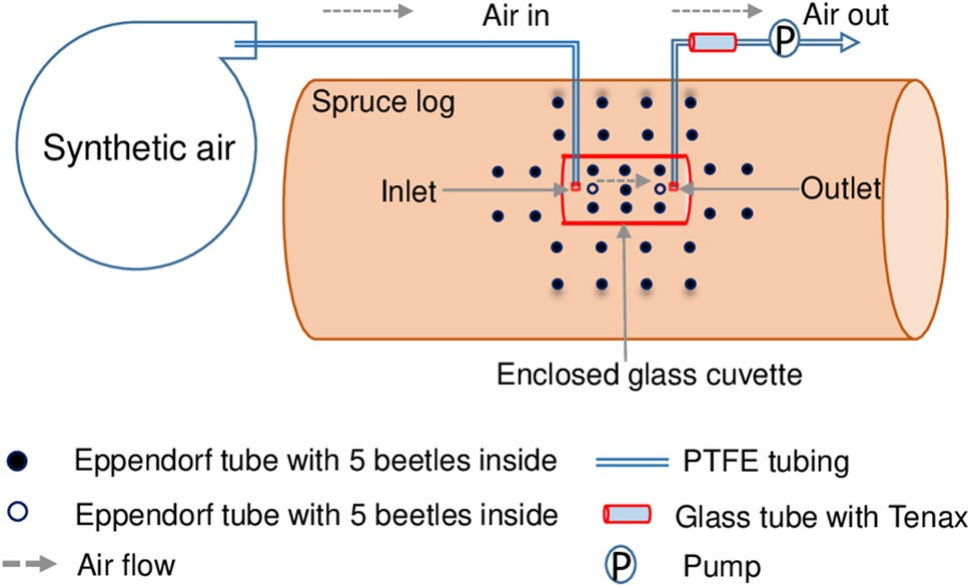
Schematic drawing of the volatile organic compound sampling system on a Norway spruce log. Synthetic air was sucked through an enclosed glass cuvette (red box) that either contained no (control), two (low-density treatment, empty dots only) or nine (high-density treatment, empty and filled dots) Eppendorf tubes. Each tube contained five live *Ips typographus*. In the high-density treatment, additional tubes with beetles were also placed around the cuvette (filled dots) to increase intraspecific competition. Air flow through the cuvette was achieved by air sampling pumps (220–1000 TC, SKC Inc. PA 15330, USA) at a constant flow rate of 200 ml min^−1^. Volatiles from 12 L cuvette air were trapped with adsorption tubes filled with Tenax and subsequently analysed via GC–MS

Alpha-pinene, verbenol and verbenone emitted from the logs were sampled with a self-designed cuvette system (Fig. 1). The customised semi-round cuvettes (inner diameter = 8.3 cm, length = 15.0 cm) made of transparent borosilicate glass (Kummer, Freiburg, Germany) with an apical inlet and an outlet (2.5 cm distant from each side) were installed and tightly sealed on the stem surface with plastic sealing band (Teroson, Henkel AG & Co. KGaA, Düsseldorf, Germany), and care to not damage the bark surface. The flat area inside the cuvette was 1.1 dm^2^ and covered the focal area with (i) 18 free pins without tubes and beetles, (ii) 2 pinned Eppendorf tubes with a total of 10 beetles plus 14 free pins, and (iii) 9 pinned Eppendorf tubes with a total of 45 beetles, for control, low and high-density treatments as well as controls, respectively (Fig. 1).

An air sampling glass tube (Gerstel, Mülheim a.d.R., Germany) filled with Tenax TA (Sigma Aldrich, Munich, Germany) was placed between the cuvette outlet and an air sampling pump (220–1000 TC, SKC Inc. PA 15330, USA) to accumulate the emitted compounds. The inlet of the glass cuvette was connected to a bag (Nalophan, Gustav Ehlert GmbH & Co. KG, Verl, Germany) containing a reservoir of synthetic air (Messer, Bad Soden, Germany) to minimise possible contamination by volatiles in the atmosphere of the greenhouse. Sampling of emitted compounds lasted for 60 min at a flow rate of 200 ml min^−1^; thus, 12 L cuvette air were passed through the air sampling tube. To ensure chemical inertness, all parts of the cuvette system were made of glass, PFA or PTFE. At day 0 of the experiment, volatiles were sampled in the morning (ca. 10:30–12:00) before and then two hours after beetles were released into the tubes (ca. 13:30–15:00). Thereafter, the sampling was done between 11:00–13:00 at daily intervals until day 4, three times in the second week, and then twice a week until the end of the experiment. In case condensed water was observed in the cuvettes, they were dried with tissue paper before sampling. After each sampling, the cuvettes were opened to ambient air. The air sampling tubes containing volatile compounds were stored at 4 °C until analysis.

### Volatile analysis with GC–MS

Alpha-pinene, verbenol and verbenone were analysed on a gas chromatograph (GC, model 7890, Agilent Technologies Böblingen, Germany) connected to a mass-selective detector (MSD, 5975C, Agilent Technologies Böblingen, Germany) and equipped with a thermodesorption/cold injection system (TDU-CIS4, Gerstel, Germany) following the method described in Kreuzwieser et al. (2021). Briefly, volatile compounds were desorbed from the adsorbent tubes by heating them up to 220°C in the TDU. Thermodesorbed volatiles were directly channelled into the CIS4 where they were cryotrapped at -70°C. Subsequently the CIS4 was heated to 240°C, releasing the volatiles onto the separation column (DB-5 ms UI, 60 m × 0.25 mm × 0.25 μm film thickness, Agilent Technologies Böblingen, Germany). Helium was used as a carrier gas at a flow of 1 ml min^−1^. The GC oven and MSD conditions as well as identification and quantification procedures followed Kreuzwieser et al. (2021). The MSD ran at 70 eV at an ion source temperature of 230°C and a quadrupole temperature of 150°C. As standards, different amounts of α-pinene ((±)-α-pinene, purity 98%), ((1*S*,2*S*)-(+)-*cis*-verbenol, 95%) and ((1*S*)-( −)-verbenone, 94%) (all standards from Sigma-Aldrich, Munich, Germany) ranging from 50–300 ng were analysed in the same manner for identification and quantification according to linear regression calibration curves (all correlation coefficients r^2^ > 0.97). The column used for GC–MS analysis was not enantioselective, so the amount of α-pinene reported is a sum of (+)- and (–)-α-pinene. β-pinene could be detected but was neglected in this study. *Trans*- and *cis*-verbenol co-eluted due to the GC program used. The mass spectra were analysed with the MassHunter Software (Agilent Technologies, Böblingen, Germany).

### Data processing and statistical analyses

Emission rates of α-pinene, verbenol and verbenone (ng dm^−1^ bark surface h^−1^) were calculated based on the volume of the sampled air, mass of the compound in this volume and bark surface area in the enclosure; additionally, we standardised the emissions by the number of beetles inside a cuvette to obtain the average release rate per beetle individual. For this purpose, the emission of the respective control log was subtracted from the total emission rate of a sample to account for non-beetle caused background emission, and then divided by the actual number of beetles still alive under the cuvette. For this part of the analysis, only the data until half of the second week (until day 11) was included because we deemed the reliability of the vitality status of the beetles too uncertain after that point (i.e., dead beetles inside the phloem were not detectable without damaging the bark). This also seems justified as most of the emission dynamic actually occurred within the first week (day 2–7, see result section).

One-way ANOVA followed by the Tukey’s post-hoc test was employed to examine the effects of density treatments on the emission rates of α-pinene, verbenol and verbenone, as well as on their ratios and standardised amounts per beetle, separately for each time point. Raw data were log10-transformed to satisfy the assumption of normal distribution. If the transformation did not lead to a better resemblance to normality, non-parametric Kruskal–Wallis One-Way Analysis of Variance on Ranks followed by the Dunn's test were employed instead. SigmaPlot 12.0 (Systat Software GmbH, Erkrath, Germany) was used for data visualisation and One-way ANOVA analysis.

To test the effect of the fluctuating temperature conditions in the greenhouse chamber, the correlation was tested between average temperature during the sampling interval (plus a 30-min buffer previous to the sampling) and the emission rate of a compound (α-pinene, verbenol, verbenone), using the non-parametric Kendall’s rank correlation τ. This analysis was done in R (R Core Team 2018) using R-Studio (RStudio Team 2022). Linear-mixed models using the *lme4*-package (Bates et al. 2015) and *lmerTest*-package (Kuznetsova et al. 2017) were fitted to assess the interaction of temperature and treatment, with the tree individual as the only random factor. A square root-transformation of the response was applied to α-pinene-concentration data, while a log(x + 0.01) transformation was used for verbenol and verbenone to satisfy the assumption of homoscedasticity. Visualisations were done with the *ggplot2*-package (Wickham 2016).

## Results

As expected, logs from the low-density group had a lower colonisation density compared to the high-density logs: After ~ 20 h (day 1) we observed boring dust in 1.8 ± 0.5 and 7.8 ± 1.3 tubes (placed within cuvettes) in the low and high-density treatment, respectively. At day 4, these numbers increased to 2.0 ± 0.0 and 8.4 ± 0.9 sites with boring dust. At the end of the experiment (day 49) there were 1.5 ± 0.6 and 3.8 ± 1.6 maternal galleries inside the measured bark area (1.1 dm^2^) for the low and high-density treatment, respectively; and 14.3 ± 6.8 vs. 24.0 ± 4.2 larval ducts. In the low-density treatment 35 ± 25% of the phloem area was consumed, while this was 50 ± 16% in the high-density treatment.

**Fig. 2:**
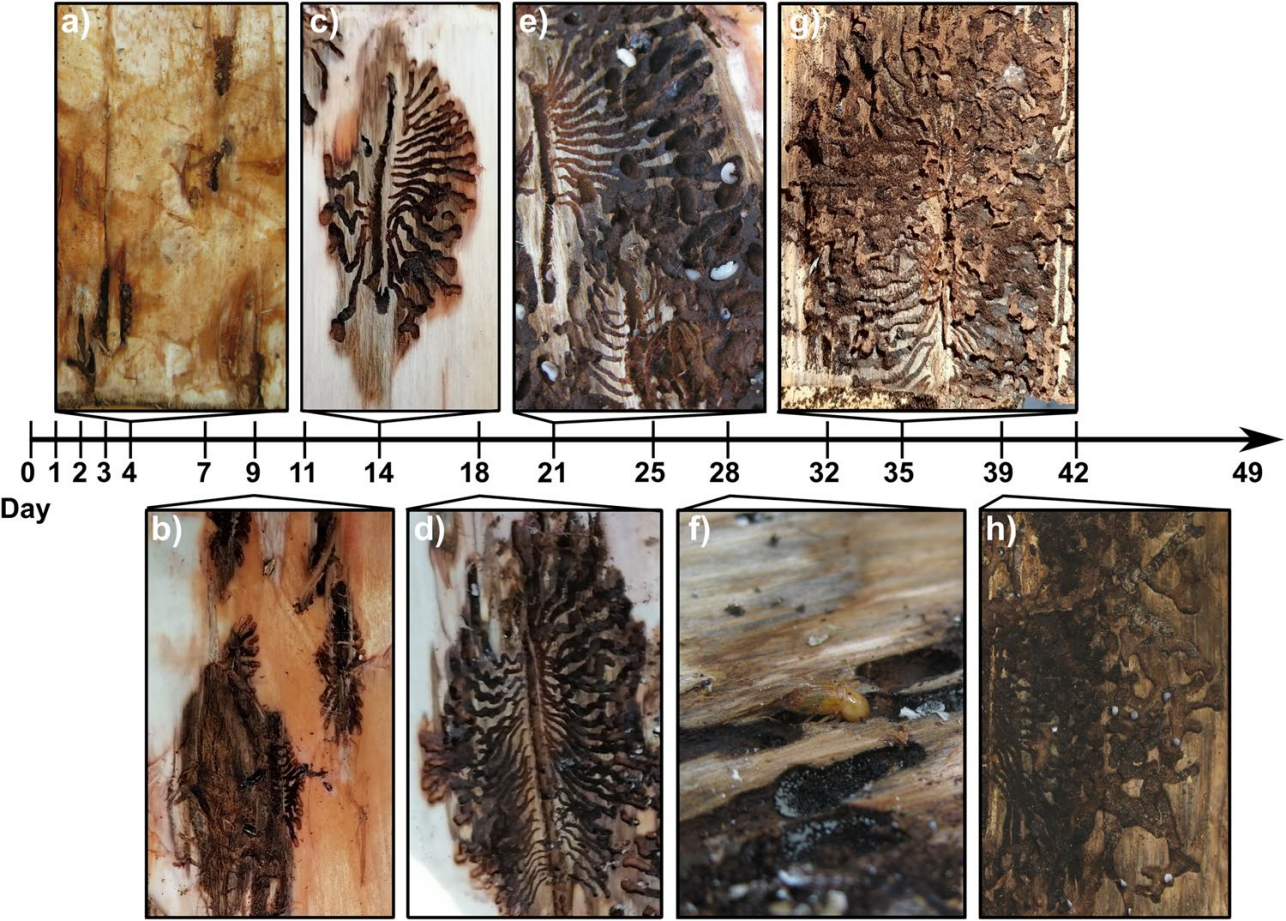
Development of *Ips typographus* over the course of the experiment as observed on the reference logs. **a** Short maternal galleries with egg niches (i.e., early oviposition); **b** early larval stage; **c** intermediate larval stage; **d** first pupal chambers; **e** pupae and first signs of body structures; **f** first callow beetles; **g** maturation feeding; **h** first beetle emergence and exit holes

Oviposition occurred at day 4 on the reference logs (Fig. 2). At day 9, the first larvae were observed. The first pupal chambers were visible at day 18 and the first pupae appeared at day 21. The first callow beetles enclosed from pupae after day 28, followed by intense maturation feeding, especially in the high-density group, which continued until the end of the experiment. The first beetles began to emerge from the logs at day 39.

**Fig. 3:**
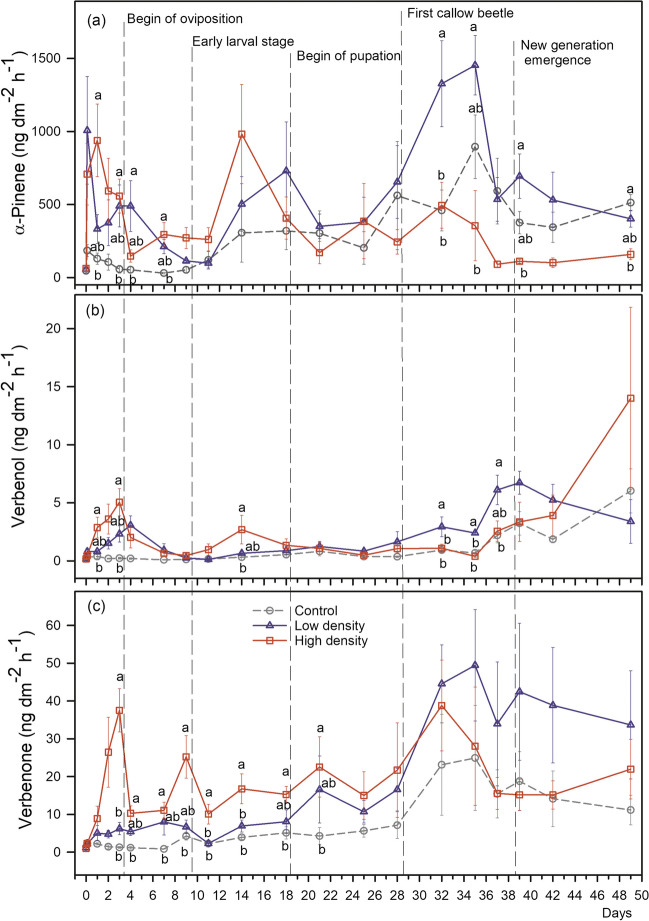
Emission rates (ng dm^−2^ h^−1^) of α-pinene (**a**), verbenol (**b**) and verbenone (**c**) from Norway spruce (*Picea abies*) logs with high (square, red) and low (triangle, blue) bark beetle (*Ips typographus* L.) colonisation densities, and negative controls without beetle colonisation (circle, black) over time. Means ± SE (n = 4–5) are shown before and directly after beetles were released (day 0), and in the following weeks (day 1–49) until the filial generation started to emerge from the brood system. Different letters indicate significant differences (p < 0.05) between high and low-density and control treatments within the same day

The emission rates of α-pinene ranged from 31 ng dm^−2^ h^−1^ in the controls to 1453 ng dm^−2^ h^−1^ in the low-density treatment. Immediately after the beetles were released, significantly higher α-pinene emission rates were observed compared to control logs (Fig. 3a). After day 7, α-pinene emissions did not differ among the treatments anymore. In the low-density treatment, α-pinene emission was significantly higher than in the control on days 32 and 35. Emission of α-pinene was weakly correlated with the average temperature during the volatile sampling (Kendall’s rank correlation τ = 0.12, p < 0.01), the interaction of treatment and temperature was not significant for the high-density group (t = -1.88, p = 0.06) (Fig. S1).

The verbenol emission rate varied from 0.1 ng dm^−2^ h^−1^ to 14.0 ng dm^−2^ h^−1^ in the control and high-density group, respectively. Verbenol emission amounted on average to only 0.1% of the α-pinene release in the control and low-density group and 1.5% in the high-density group. A steady increase in verbenol emission was observed in the high-density treatment from day 1 with a peak at day 3 (Fig. 3b). The emission pattern was similar to the low-density treatment, where the verbenol emission rate peaked one day later (day 4). Verbenol emission then declined to the level of the control after day 7 in both treatment groups. During day 1–3 verbenol emission was 5.8 times greater in the high-density treatment compared to the control and 2.5 times greater than in the low-density treatment. On day 4, verbenol emission was 1.5 times higher in the low-density treatment compared to the high-density treatment and 15.3 times higher than in the control group. A second peak of verbenol emission was observed at day 14 during the larval stage in the high-density treatment (p = 0.01). Significantly higher verbenol emission rates were observed in low-density treated logs at days 32, 35 and 37. The highest verbenol emission was documented at day 49 in the high-density treatment, however, the differences between different treatments were not significant (p = 0.20) due to the large variations (Fig. 3b). Verbenol emission hardly depended on temperature (Kendall’s rank correlation τ = 0.06, p = 0.17) and the temperature effect did not interact with the treatment (p > 0.14 for all interaction terms) (Fig. S2).

Verbenone emission ranged from 0.8 ng dm^−2^ h^−1^ to 49.5 ng dm^−2^ h^−1^ in the control and low-density group, respectively. It amounted on average to 4%, 5% and 19% compared to the α-pinene emission, in the control, low and high-density group, respectively. Verbenone emission rates in the high-density treatment were significantly higher compared to control logs from day 3 until day 21 (0.001 < p ≤ 0.03; Fig. 3c). Also on day 2, the average verbenone emission in the high-density treatment was 18 times greater compared to the control but this difference was statistically not significant (p = 0.14). From day 2 until day 21 verbenone release in the high-density treatment was on average 7 times greater than in the control group. Even though not significant (0.12 < p < 1.00), also the verbenone release from the low-density group was on average 2.6 times greater compared to the control from day 2 until day 21. After three weeks, there were no significant differences between the treatments and control logs. Verbenone emission was significantly correlated with temperature (Kendall’s rank correlation τ = 0.19, p < 0.01) with the temperature effect not interacting with the treatment (p > 0.16 for all interaction terms) (Fig. S3).

**Fig. 4:**
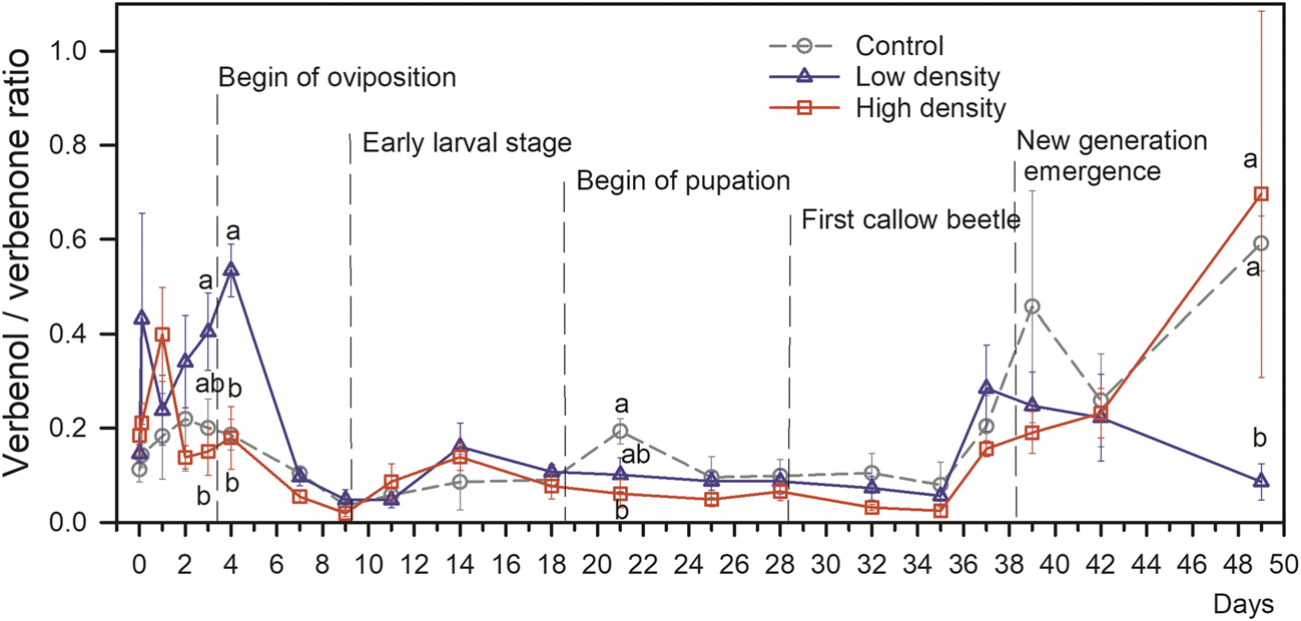
Ratios between verbenol and verbenone emission rates of Norway spruce (*Picea abies*) logs with high (square, red) and low (triangle, blue) bark beetle (*Ips typographus* L.) colonisation density, and negative control without beetle colonisation (circle, black) over time. Means ± SE (n = 4–5) are shown before and directly after beetles were released (day 0), and in the following weeks (day 1–49) until the next generation started to emerge. Different letters indicate significant differences (p < 0.05) between high and low-density treatments and controls within the same day

Verbenol to verbenone emission ratio ranged from < 0.01 to 2.23 as observed in the high-density treatment at days 9 and 49, respectively (Fig. 4). From day 2 to 4, the verbenol to verbenone ratio was significantly higher in the low-density (0.43 ± 0.17) compared to the high-density logs (0.16 ± 0.11) and the control logs (0.20 ± 0.14), the latter two being non-significantly different. After day 4, the verbenol to verbenone ratio remained relatively constant until the end of the experiment except the last day (Fig. 4).

The emission rates of the three compounds were all significantly (p < 0.01 in all cases) and positively correlated. The highest correlation was found between verbenol and verbenone emissions (Kendall’s rank correlation τ = 0.47), and the lowest correlation between α-pinene and verbenone (τ = 0.33). The correlation coefficient was intermediate for α-pinene and verbenol (τ = 0.45).

**Fig. 5:**
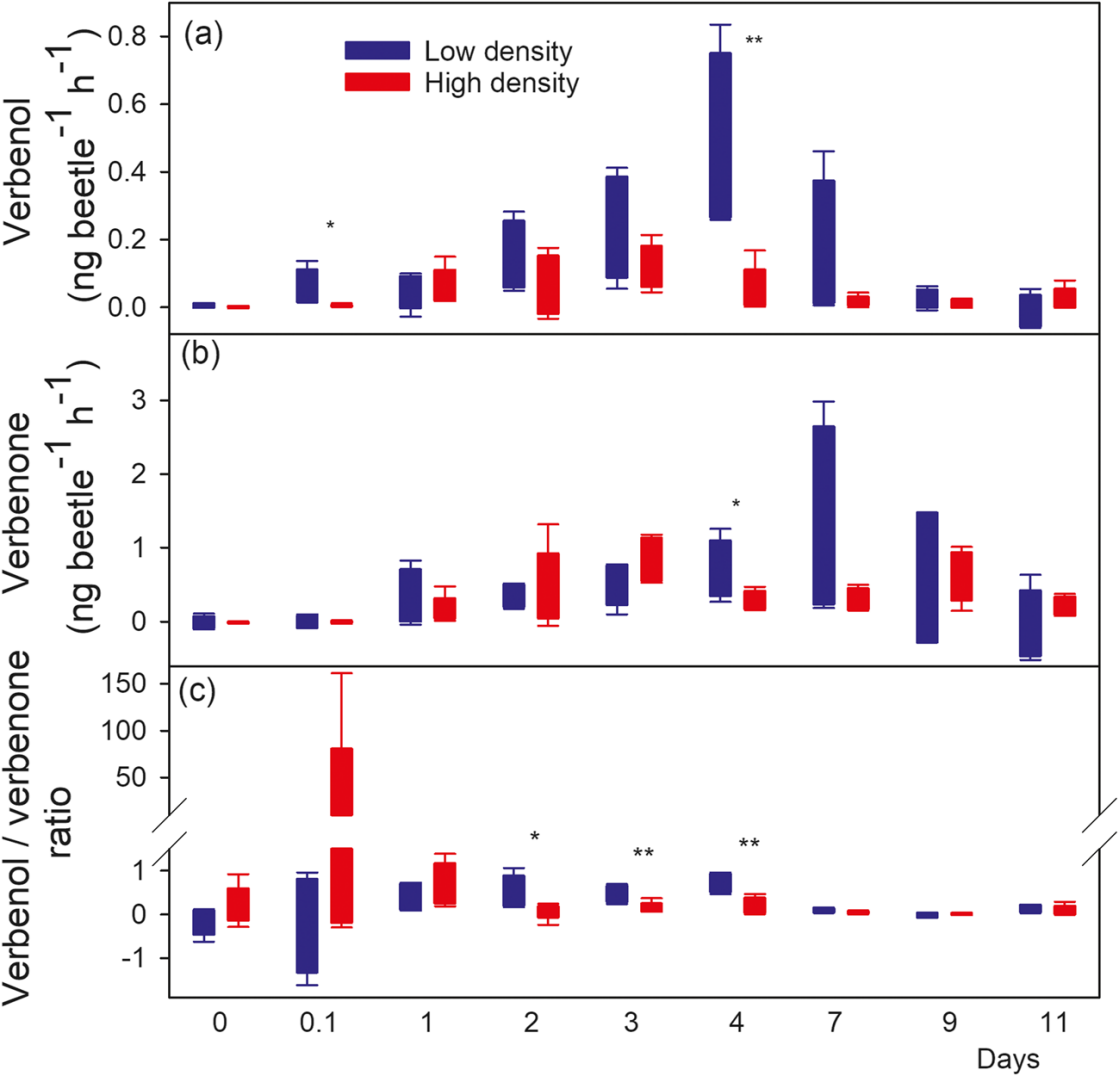
Average emission of verbenol (**a**) and verbenone (**b**) per beetle individual colonising Norway spruce (*Picea abies*) logs as well as their ratios (**c**) at high (red) and low (blue) bark beetle (*Ips typographus* L.) colonisation densities. Shown are the means ± SE (n = 4–5) of the emission rate of each compound during the first 1.5 weeks after host colonisation. The asterisks refer to the significance thresholds of *: *p* < 0.05 and **: *p* < 0.01

The emission rates of verbenol and verbenone, as well as their ratios, were also standardised by the number of beetles to compare the average emission rate on the level of individuals after accounting for the background emission from pure bark without beetles as observed on the reference logs (Fig. 5). The verbenol to verbenone ratio was significantly greater in the low-density treatment compared to the high-density treatment on days 2–4 (p < 0.05) and still 1.7 times but not significantly greater on day 7 (p = 0.11, Fig. 5c). During that period (days 2–7), the verbenol to verbenone ratio was on average 0.48 ± 0.22, and 0.24 ± 0.17 in the low and high-density treatment, respectively.

During days 2–7, per-beetle verbenol emission was 0.25 ± 0.22 ng beetle^−1^ h^−1^, and 0.06 ± 0.07 ng beetle^−1^ h^−1^ in the low and high-density treatment, respectively (Fig. 5a). This difference was significant on day 4 (p = 0.01), but not significant on the other days (p ≥ 0.09) when verbenol emission per beetle was on average 2.5 times greater in the low compared to the high-density treatment. On all other days the differences were not significant (p ≥ 0.18), with the exception of day 0.1 (i.e., shortly after introducing the beetles on the logs), when the low-density treatment also showed significantly higher verbenol emission per beetle (p = 0.04). The verbenone emission per beetle during days 2–7 was 0.72 ± 0.72 ng beetle^−1^ h^−1^ and 0.51 ± 0.39 ng beetle^−1^ h^−1^ in the low and high-density treatment, respectively. On day 4, verbenone emission per beetle was significantly higher in low compared to high-density treatment (p = 0.04). It is important to note, that the trend in the means, unlike for verbenol, was not consistent throughout this period for the emission of verbenone. Instead, on days 2–3 the emission was slightly greater from the high-density treatment and this changed for days 4 and 7.

## Discussion

Verbenone has been proposed as an aggregation inhibitor in several bark beetle species to reduce intraspecific competition (Borden 1997; Vité and Francke 1985; Bakke 1981, 1987; Schlyter et al. 1989; Unelius et al. 2014; Zhang et al. 1999). However, data on the natural occurrence of verbenone did not unambiguously support this putative function for *I. typographus* due to its early occurrence during host colonisation (Birgersson et al. 1984; Birgersson and Bergström 1989). Our study provides first evidence that i) verbenone emission is associated with bark beetle colonisation density, and ii) occurs during a relevant time span to function as an aggregation inhibitor during host colonisation and brood development.

In accordance with our initial hypothesis, verbenone emission was clearly related to bark beetle colonisation density. We observed that an initial 4.5 times higher infestation density causes a 2–3 times increase in verbenone emission during the three weeks lasting period of larval development, and 3–7 times increased emission compared to non-infested logs. To our knowledge, the only other study considering an equally large time span did not find significant differences in verbenone emission between logs infested with *I. typographus* and non-infested logs (Pettersson and Boland 2003). However, the colonisation density was not reported in that study and may have been too low to induce enhanced verbenone emission. In our experiment, even the lower infestation density caused around 3 times greater verbenone emission compared to non-colonised logs, but these differences were not significant on most days because of the large variation.

During the time of larval development until pupation, the verbenone emission amounted to 7–34 ng dm^2^ h^−1^ from bark colonised at densities of 1.8–8.4 bore holes dm^−2^. Assuming a bark surface of roughly 10 m^2^ on a suitable Norway spruce host (diameter at breast height: ~ 28 cm, height: ~ 25 m), this would result in an emission of 175–815 µg d^−1^ at the whole-tree level. This value lies more than ten times below the estimated 10,800 µg d^−1^ possibly released from a tree infested with 1,400 pairs of *D. frontalis* (Salom et al. 1992). In spruce ecosystems, male *I. typographus* bore hole density varies from 0.4–1.0 holes dm^−2^ on naturally infested logs or windthrows (Anderbrant et al. 1988; Bakke 1987; Toffin et al. 2018) to densities of 2.33 holes dm^−2^ (Toffin et al. 2018) and even 5 holes dm^−2^ are possible (Paynter et al. 1990; and pers. obs.). We have chosen the high-density treatment to simulate an extremely high – and rather unnatural – colonisation density in order to ensure that there is a significant level of intraspecific competition from the outset. While our low-density treatment resulted in a 4.5 times lower initial colonisation density compared to the high-density treatment (the resulting number of maternal galleries was around 2.5 times lower at the end of the experiment), it rather reflects a medium to high density under natural conditions. In that regard, our extrapolated whole tree verbenone emission rates rather describe the upper range of what would be expected under natural conditions.

It is surprising, however, that contrary to our hypothesis verbenone emission increased as early as on day 1 for the high beetle density, and on day 2 for the low beetle density. Yet, this finding is well in accordance with other studies on *I. typographus* (Birgersson et al. 1984; Birgersson and Bergström 1989) and other bark beetle species that are also inhibited by verbenone (e.g. Byers et al. 1984; Shi and Sun 2010; Xie and Lv 2012). The observed early emission of verbenone seems to contradict its assumed role as an aggregation inhibitor to reduce intraspecific competition at later stages of colonisation. It takes about 1–2 days for the males to finalise the nuptial chamber (Birgersson et al. 1984). During this time, the colonising males need to attract mating partners to the tree, and, in case of a strong defence by the host tree, also conspecific males. The unexpected early appearance of verbenone might be caused from its production during the metabolization of host-derived α-pinene (Frühbrodt et al. 2023; Hunt and Borden 1989).

Another rather unexpected aspect is the drop of verbenone emission to rates similar to logs without beetles, after 21 days in our experiment, coinciding with the termination of larval development. If the bark beetles employ verbenone as a cue for unsuitable hosts (Lindgren and Miller 2002), then this cue should still be relevant because the breeding conditions continuously decrease with ongoing beetle maturation. Interestingly, the observed period of verbenone emission is comparable to the release of other host-marking pheromones in phytophagous insects (Liu et al. 2011). Studies on other insects suggest that most host-marking pheromones only occur during the most critical time spans and disappear when, for example, the offspring already has a head-start ensuring its competitiveness (Nufio and Papaj 2001). Moreover, the low availability of precursor compounds (e.g. verbenol emission was on the level of no-beetle control logs), indicates that the production of verbenone might have been limited at this time. In addition, a cessation with ongoing pupation suggests that larvae and/or larval feeding may be at least partly responsible for the emission of verbenone (Grégoire et al. 1991) although volatile emission from larvae is not well studied in bark beetles so far.

We did not find evidence that the verbenone emission is actively controlled by the beetles because the per-beetle emission of verbenone did not differ consistently between the two density treatments (Fig. 5). Yet, the verbenone emission per bark area was significantly greater with greater colonisation density. Therefore, verbenone emission strength is most likely an additive effect, i.e., more beetles emit more verbenone, but without colonisation density-dependent up-regulation at the level of individuals. The amount of verbenone that is emitted thus indicates the number of beetles already present on a host. It is not uncommon that insects are capable of assessing the number of conspecifics already present at an oviposition site and subsequently face a decision that is not a clear yes-or-no but a weighing-up decision whether to lay eggs or not (Nufio and Papaj 2001; Renwick 1989).

The finding that *I. typographus* may not be able to regulate the emission of verbenone supports the assumption that its biosynthesis is mainly achieved by associated microorganisms inside and outside the beetles (Leufvén et al. 1988). There are well described examples of insects actively controlling their internal microbiome (Whittle et al. 2021), as well as their surrounding microbiome (Diehl et al. 2022) helping the beetles to succeed in colonizing the trees by modifying their substrate and improving nutrition. Nevertheless, a strong mutual selection would be necessary for such a relationship. The most parsimonious interpretation in our case is to assume that verbenone is mostly synthesised by associated microorganisms, e.g. ophiostomatoid fungi (Biedermann et al. 2019; Cale et al. 2019; Kandasamy et al. 2023) and yeasts (Hunt and Borden 1990; Leufvén et al. 1984) without a direct or very strong control of the beetles. Consequently, verbenone appears like a passive, reliable cue of high beetle density and associated microbial activity inside a tree allowing newly arriving beetles to differentiate between a densely colonised and a decaying but not yet colonised host.

*Cis*-verbenol is one of the three known aggregation pheromone components of *I. typographus* (Schlyter et al. 1987a, b). Consequently, we hypothesised high release rates during the initial phase of host colonisation when conspecifics are required for overcoming host defence of living trees, and mating (on dead and living trees) (Raffa 2001). Accordingly, our data suggests that verbenol does behave like an ideal aggregation pheromone: its emission rate increased after day 1 and was only present in significant amounts until day 4 (early oviposition) at both infestation densities. After day 4, verbenol emission declined to the level of logs without beetles. The observed decline of verbenol after the initial phase of host colonisation is in accordance with other studies conducted on hindgut extracts (Birgersson et al. 1984), individual bore holes (Birgersson and Bergström 1989) and the phloem around bark beetle galleries (Leufvén and Birgersson 1987). It has to be mentioned, that verbenol consists of two isomeric forms, *cis*- and *trans*-verbenol, of which only *cis*-verbenol is behaviourally relevant in *I. typographus* (Renwick et al. 1976; Schlyter et al. 1987a). Unfortunately, our analytical setup did not allow differentiation of the two isomers. Both *cis*-verbenol and *trans*-verbenol are produced from the oxidation of α-pinene but (1*S*,2*S*)-( +)-*cis*-verbenol is selectively formed from (–)-α-pinene according to the literature (Klyne and Buckingham 1974; Lindström et al. 1989; Renwick et al. 1976). Both verbenol isomers can be transformed to verbenone (Leufvén et al. 1984; Lindmark-Henriksson et al. 2003). Noteworthy, in our study, verbenol and verbenone emissions significantly correlated to a similar degree as the emissions of α-pinene and verbenol. In conclusion, even though we could not reveal the behaviourally relevant proportion of *cis*-verbenol, we are confident that we succeeded in showing the link between verbenol and verbenone emission to the colonisation density of *I. typographus* over time. The resulting signal that is conveyed and the actual beetle behavioural response then depends to a certain degree on the host and its enantiomeric composition of α-pinene. It would be interesting to assess the role of *cis*-verbenol in *I. typographus* in relation to the host tissue quality and primary attraction in future studies. Possibly, knowledge on such patterns would help to explain why some trees seem less likely to be colonised by bark beetles, in case the beetles are not able to synthesise their pheromone component.

The observed patterns of verbenol and verbenone emissions in our study support the “quantitative hypothesis” of Schlyter et al. (1987b) that the shift of landing beetles from a focal tree to an adjacent and less densely colonised trees is caused by an interplay of two mechanisms: i) a cessation of the release of aggregation components (verbenol) and ii) a release of aggregation inhibitors (verbenone). This sufficiently long release of an aggregation inhibitor like verbenone was postulated by Schlyter et al. (1987b) but not proven; our data fully support their hypothesis. It is speculative if the density regulation within a tree and the propagation of colonisation to adjacent trees may additionally be enhanced by a sex-specific response in a way that male *Ips* are inhibited by high amounts of aggregation pheromone, while females are still attracted (Byers 1983).

Bark beetles are well known for their quite pronounced synchronisation during swarming and colonisation (Sauvard 2004). Yet, host colonisation under natural conditions is a process of several weeks (Anderbrant et al. 1988; Toffin et al. 2018), or even longer (Göthlin et al. 2000). According to our experimental design, host colonisation was strongly synchronised within one day, allowing us to detect relatively clear temporal emission patterns for verbenol and verbenone over time as described above. The theoretical outcome of a delayed colonisation is an overlap of the volatile emission patterns from groups of individual beetles arriving at different moments (Fig. S4). This may result in synchronous phases of high volatile emission that are interrupted whenever the colonisation process is also inhibited, e.g. due to unfavourable weather conditions (see Birgersson and Bergström 1989). These ‘pulses’ of volatile emission and the resulting effect on beetle attraction and colonisation may ultimately lead to quite regular colonisation pattern within a tree, that occurs when newcomers gradually fill the gaps on the bark among sites already occupied by conspecifics (Toffin et al. 2018).

It was surprising to find verbenol emission re-appearing in significant amounts from logs infested at lower density when the first callow beetles appeared in the brood system, because at this point an aggregation signal would be pointless for newly arriving beetles. The re-appearance of verbenol with the onset of maturation feeding, however, may emphasise the evolutionary origin also of verbenol from the oxidative metabolisation of α-pinene (Fang et al. 2020; Leufvén and Birgersson 1987). It would also explain why we did not find increased verbenol emission from logs with higher infestation density at the same time, for which the emission of α-pinene decreased to a level even below the level of logs without beetles. We assume that the majority of α-pinene in that bark area had largely been depleted by then, as also supported by the 2.5 times higher consumed phloem area in the high-density treatment. The occurrence of verbenol associated with callow beetles still on their brood tree is in accordance with the findings by Ramakrishnan (2022), who detected large amounts of verbenol in gut extracts from male and female *I. typographus* before emergence. Previously it had been assumed that no attraction is elicited at this stage because other synergising cues and signals are likely missing (Schlyter et al. 1987a), but given that rates of pre-emergence matings are very high in *I. typographus* (Dacquin et al. 2023) it is possible that verbenol serves the attraction of mates already inside the brood tree. There was also an increase of verbenol emission in the control group after day 36. Even intact plant tissue is not sterile, but colonised by microorganisms (Sieber 2007). Together with other microorganism that likely entered the bark due to pinning (see methods), this may have caused the increased emission also from non-colonised logs.

Contrary to verbenone, the per-beetle emission of verbenol was consistently and on average 4 times greater from individuals of the low-density treatment compared to individuals colonising at high densities during the initial phase (i.e., the first week) after colonisation. This was also reflected by the verbenol to verbenone ratio on logs infested at a low density that remained high until day 4, while it dropped already after the first day in the high-density group. Both findings suggest that the emission of verbenol and verbenone are not merely proportionally linked to the number of beetles within a given bark area. Instead, their ratio is obviously affected by the colonisation density. Since the per-beetle emission of verbenone is not affected by the density of colonising beetles, it is the verbenol emission that causes this changed ratio. Wallin and Raffa (2002) observed that the density of conspecifics in *I. pini* already present in artificial phloem media significantly affected the probability and speed of gallery construction, indicating that bark beetles are somehow capable of assessing the local beetle density and respond accordingly. In *Dendroctonus pseudotsugae* and *D. valens* acoustic signals from the males cause emission of anti-aggregation pheromones (Liu et al. 2016; Rudinsky 1969), but similar mechanisms are not yet described for *I. typographus*. Possibly verbenol can be emitted by beetles in a controlled way, because *I. typographus* can store *cis-*verbenol in the fat body as verbenyl oleate (Ramakrishnan 2022).

It was remarkable, that the strongest emission from the infested logs consisted of the untransformed verbenol/verbenone precursor α-pinene (≥ 80%), which is among the most abundant monoterpenes emitted from Norway spruce (Duan et al. 2020). The emission of α-pinene, as well as verbenol and verbenone showed large variations within treatments, as also observed by other studies (e.g. Birgersson et al. 1988; Birgersson and Bergström 1989; Ghimire et al. 2016; Leufvén and Birgersson 1987; Schiebe et al. 2019), at least partly due to the genetic variations of logs from different tree individuals, as well as assumed differences in the resistance for volatile emission due to differences in bark thickness (Lindström et al. 1989) in the present study. The emission also showed a rather variable pattern over time as also observed in other studies (e.g. Birgersson and Bergström 1989). Compared to verbenol and verbenone, the emission of α-pinene depended more strongly on air temperature (Figs. S1-S3), suggesting that a large proportion was passively released from the logs. Nevertheless, as hypothesised, its emission was also clearly linked to beetle colonisation, as indicated by the strong increase in emission upon infestation by the beetles and the resulting production of frass, as well as the significant interaction of temperature and colonisation density as a predictor for α-pinene emission. The emission from logs without beetle activity (negative controls) was also relatively high during later stages, presumably because of elevated temperatures since temperature during volatile sampling was on average 1.2°C greater after day 28 (when callow beetles appeared in the brood system) compared to the period before.

## Conclusions

To our knowledge, we provide first evidence that the emission of the aggregation inhibitor verbenone in *I. typographus* is linked to colonisation density and may thus serve as a cue to identify unsuitable breeding habitat and reduce intraspecific competition for a sufficiently long and relevant time span during host colonisation and brood development. Based on our findings, we think that verbenone is a passive and reliable cue indicating high colonisation density as we did not find support for an active regulation of verbenone emission by the beetles in our study. This also supports the assumption that verbenone biosynthesis is mainly achieved by associated microorganisms rather than by the beetles themselves. Our results also suggested that the emission of the aggregation pheromone component verbenol is linked to α-pinene available to the beetles and possibly is even actively regulated by the beetles as the emission from individual beetles was significantly greater during the initial phase of host colonisation when the colonisation density was low. The cessation of verbenol release after this initial phase in addition to the continued emission of verbenone likely explains the tree shifting during bark beetle colonisation, supporting the “quantitative hypothesis” formulated by Schlyter et al. (1987b). Our results add on the understanding of the colonisation dynamic of bark beetles and may thus also help to optimise verbenone-based management strategies, e.g. by adapting release rates of pheromone dispensers to natural emission rates. We encourage further studies to investigate the underlying mechanisms on density-dependent pheromone emission regulation in bark beetles, and the comparison with other groups of insects to obtain a holistic ecological and evolutionary understanding of processes comprising host selection, colonisation and regulation of intraspecific competition.

### Supplementary Information

Below is the link to the electronic supplementary material.

Supplementary file1 (DOCX 417 KB)

## Data Availability

The raw data of this study are available from the corresponding author upon reasonable request.
