# Correction: Know When You Are Too Many: Density-Dependent Release of Pheromones During Host Colonisation by the European Spruce Bark Beetle, *Ips Typographus* (L.)

**DOI:** 10.1007/s10886-024-01510-0

**Published:** 2024-05-20

**Authors:** Tobias Frühbrodt, Baoguo Du, Horst Delb, Tim Burzlaff, Jürgen Kreuzwieser, Peter H. W. Biedermann

**Affiliations:** 1 Department of Forest Protection, Forest Research Institute Baden-Württemberg, Wonnhaldestrasse 4, 79100 Freiburg, Germany; 2 Chair of Ecosystem Physiology, University of Freiburg, Georges-Köhler-Allee 53, 79110 Freiburg, Germany; 3 Chair of Forest Entomology and Protection, University of Freiburg, Fohrenbühl 27, 79252 Stegen- Wittental, Germany

**Correction: Journal of Chemical Ecology (2023) 49:652–665**

10.1007/s10886-023-01453-y.

The authors of paper “Know When You Are Too Many: Density-Dependent Release of Pheromones During Host Colonisation by the European Spruce Bark Beetle, *Ips Typographus* (L.)” published in the “Journal of Chemical Ecology”, Volume 49, pages 652-665, 2023 have found that the second dotted line indicating “Early larval stage” in Figure 3 was in a wrong position, i.e., it should have been shortly after day 9 as in Figure 4 and also as indicated in Figure 2.

The original article has been corrected.

