# Supplementary material for: Know When You Are Too Many: Density-Dependent Release of Pheromones During Host Colonisation by the European Spruce Bark Beetle, Ips typographus (L.)
Source: J Chem Ecol. 2023 Oct 3;49(11-12):652–65. doi: 10.1007/s10886-023-01453-y (PMC10781875; doi:10.1007/s10886-023-01453-y)
Supplement: Supplementary file 1 — Supplementary file1 (DOCX 417 KB) [file 10886_2023_1453_MOESM1_ESM.docx]

**Know When You Are Too Many: Density Dependent Release of Pheromones During Host Colonisation by the European Spruce Bark Beetle, *Ips typographus* (L.).**

Tobias Frühbrodt ^1^, Baoguo Du ^2*^, Horst Delb ^1^, Tim Burzlaff ^3^, Jürgen Kreuzwieser ^2^, Peter HW Biedermann^3^

^1^ Forest Research Institute Baden-Württemberg, Dept. Forest Protection, Wonnhaldestrasse 4, 79100 Freiburg, Germany

^2^ Chair of Ecosystem Physiology, University of Freiburg, Georges-Köhler-Allee 53, 79110 Freiburg, Germany

^3^ Chair of Forest Entomology and Protection, University of Freiburg, Fohrenbühl 27, 79252 Stegen-Wittental, Germany

*E-Mail of corresponding author: baoguo.du@ctp.uni-freiburg.de

**Supplementary information of manuscript submitted to the Journal of Chemical Ecology**

**Supplementary Information**


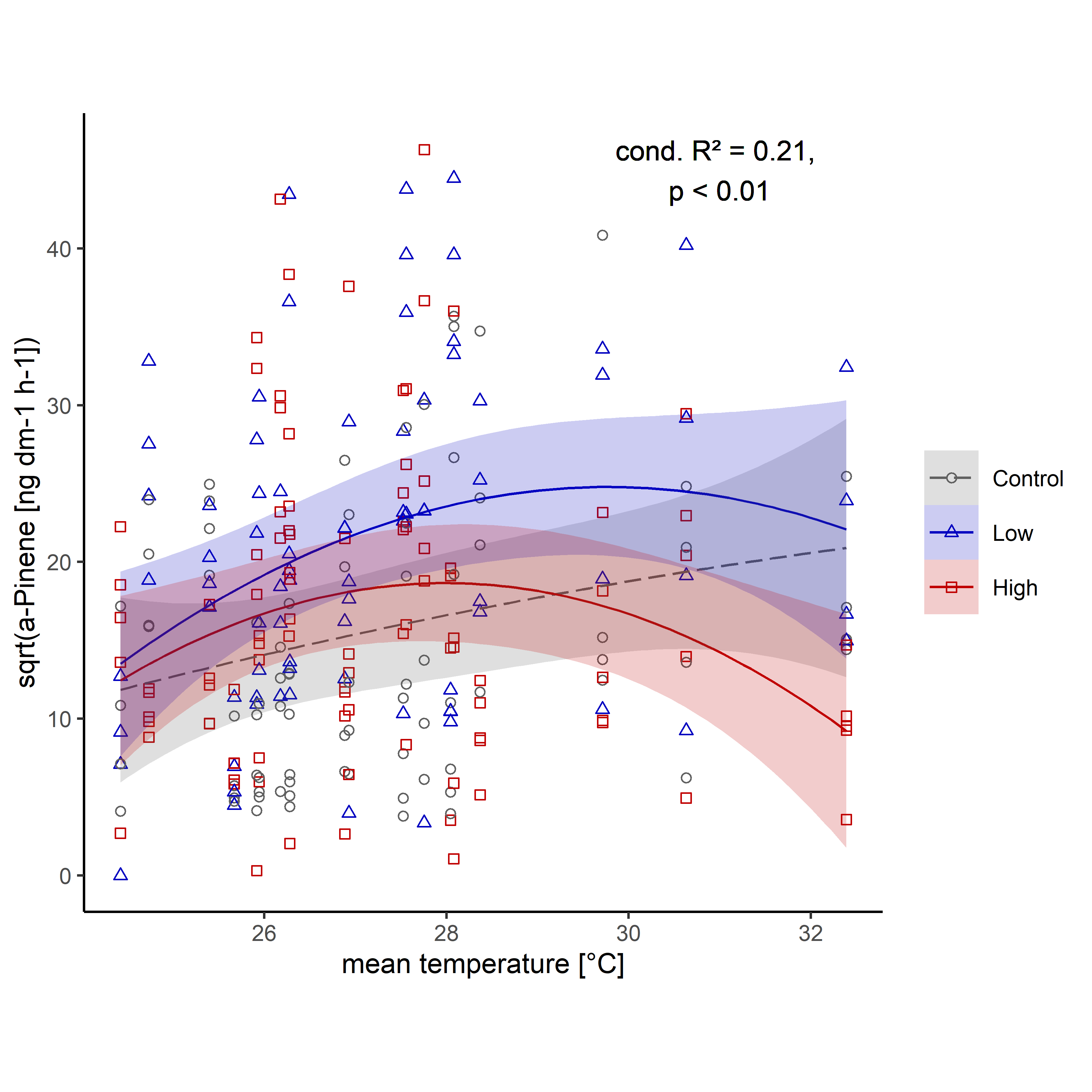


**Fig. S1** Effect of mean temperature during volatile sampling on the emission of α-pinene (square-root transformed) from Norway spruce logs with low (blue, triangle) high (red, square) and without (grey, circle) bark beetle colonisation.


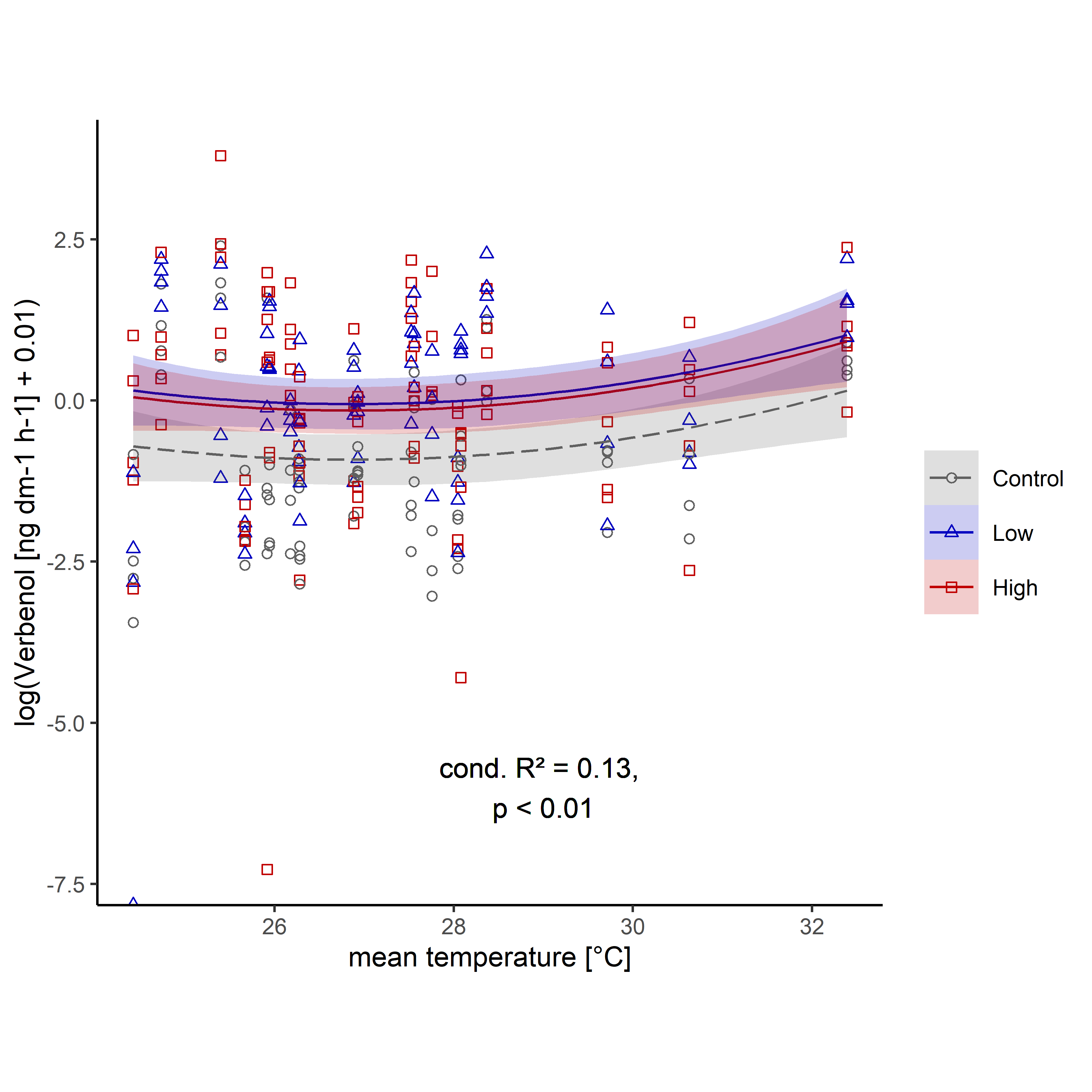


**Fig. S2** Effect of mean temperature during volatile sampling on the emission of verbenol (square-root transformed) from Norway spruce logs with low (blue, triangle) high (red, square) and without (grey, circle) bark beetle colonisation.


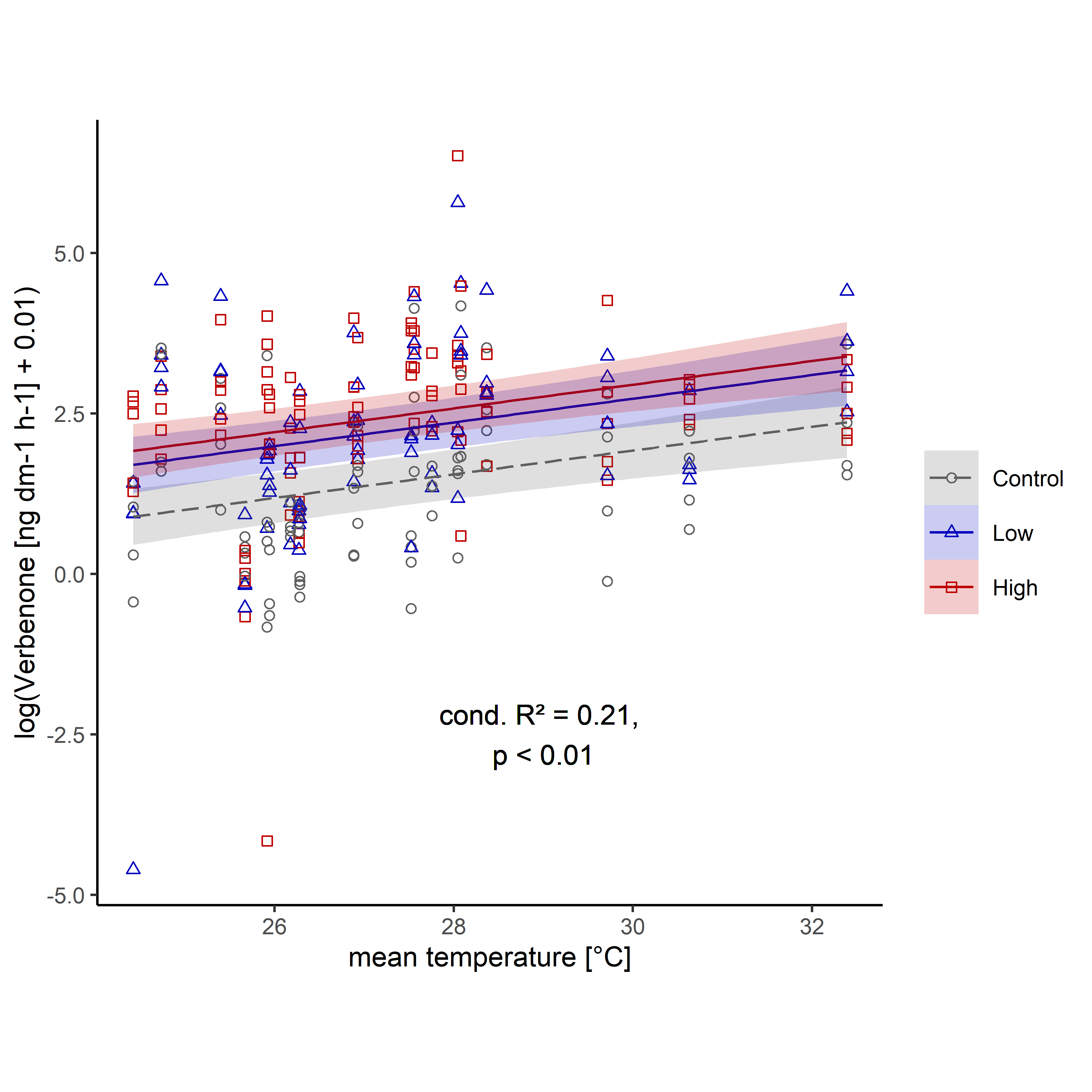


**Fig. S3** Effect of mean temperature during volatile sampling on the emission of verbenone (square-root transformed) from Norway spruce logs with low (blue, triangle) high (red, square) and without (grey, circle) bark beetle colonisation.


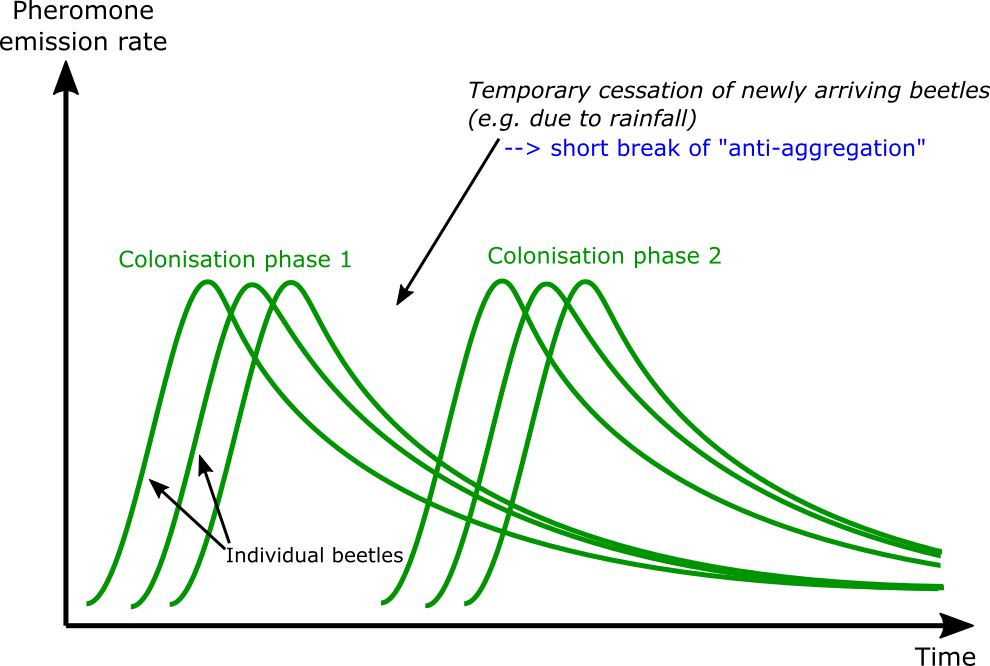


**Fig. S4** Schematic pheromone emission dynamic during host colonisation of gregarious bark beetles. Pheromone emission patterns of individuals that colonise at the same moment in time overlap, resulting in high total emission rates from a tree. Contrarily, no or lower overlap occurs for individuals that colonise with some delay. During longer breaks of the colonisation process (e.g. due to unfavourable weather conditions) the overall pheromone emission might almost cease and re-initiate in a next phase of colonisation. This example is based on an anti-attractant, but the same pattern can be expected for the emission of aggregation pheromones. Ultimately, the emission patterns of all relevant volatile compounds need to be integrated to deduce the corresponding behavioural response.
